# Changing biventricular mechanics during thrombectomy for intermediate high-risk pulmonary embolism

**DOI:** 10.1093/eurheartj/ehac508

**Published:** 2022-09-21

**Authors:** Antoon J M van den Enden, Christiaan L Meuwese, Nicolas M Van Mieghem

**Affiliations:** Department of Cardiology, Thoraxcenter, Erasmus University Medical Center, office Nt-645, Dr. Molewaterplein 40, 3015 GD Rotterdam, The Netherlands; Department of Cardiology, Thoraxcenter, Erasmus University Medical Center, office Nt-645, Dr. Molewaterplein 40, 3015 GD Rotterdam, The Netherlands; Department of Intensive Care Adults, Erasmus University Medical Center, Rotterdam, The Netherlands; Department of Cardiology, Thoraxcenter, Erasmus University Medical Center, office Nt-645, Dr. Molewaterplein 40, 3015 GD Rotterdam, The Netherlands

**Figure:**
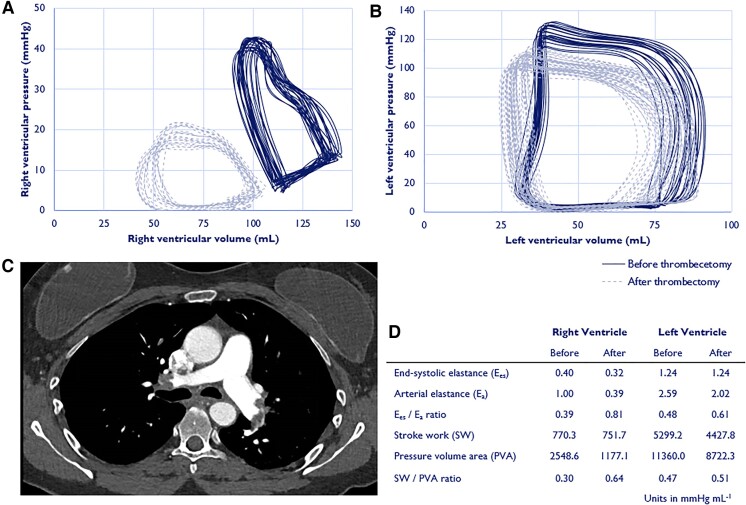


A 49-year-old female with recent intracranial bleeding was admitted after an unwitnessed syncope. On hospital admission, oxygen saturation was 97% on room air, heart rate was ≥100 beats/min, and blood pressure was 121/52 mmHg. Pulmonary embolism (PE) was confirmed by computed tomography, affecting bilateral central and subsegmental branches (*Panel C*). Transthoracic echocardiography demonstrated a dilated right ventricle (RV) with flattened interventricular septum (see Supplementary material online, *Video S1*). Troponin-T level was 133 ng/L (reference: <14 ng/L). Despite heparin therapy, blood pressure decreased upon minor mobilization. Endovascular thrombectomy was performed using the 24Fr Flowtriever (Inari Medical, Basel, Switzerland) with concomitant invasive biventricular pressure–volume (PV) loop monitoring to appraise immediate changes in cardiomechanics.^1,2^ Heart rate immediately fell from 105 to 85 beats/min. Systolic pulmonary artery pressure dropped from 42 to 19 mmHg. A prompt left-shift of the RV PV loop indicated direct RV unloading (*Panel A*). Biventricular PV area (PVA) decreased with higher stroke work/PVA ratio, suggesting improved cardiomechanics leading to enhanced myocardial metabolic demand (*Panels A, B, and D*, Supplementary material online, *Table S1*). Arterial elastance (*E*_a_) decreased for both ventricles. Right ventricle PV loop morphology transformed from notched to quadratic throughout thrombectomy (*Panel A*).^3^ Ventricular–vascular coupling (*E*_es_/*E*_a_ ratio) improved for both ventricles in the presence of stable load-independent contractility (the end-systolic elastance, *E*_es_). The patient could mobilize within hours after thrombectomy and was discharged home the next day. These observations underscore the immediate favourable effects of restored pulmonary flow on biventricular cardiomechanics in the setting of intermediate high-risk PE. The patient provided informed consent for publication.

Supplementary material is available at *European Heart Journal* online.

A.J.M.v.d.E. received personal fees from Abiomed and honorarium from Angiodynamics. C.L.M. declares no conflict of interest. N.M.V.M. received grant support from Abbott Vascular, Biotronik, Boston Scientific, Edwards Lifesciences, Medtronic, Daiichi Sankyo, Abiomed, PulseCath BV, FEops, and Pie Medical and received financial support from Antaris Medical and JenaValve.

The data underlying this article will be shared on reasonable request to the corresponding author.

## Supplementary Material

ehac508_Supplementary_Data
