# Supplementary material for: Changing biventricular mechanics during thrombectomy for intermediate high-risk pulmonary embolism
Source: Eur Heart J. 2022 Sep 21;44(11):1001. doi: 10.1093/eurheartj/ehac508 (PMC10011330; doi:10.1093/eurheartj/ehac508)
Supplement: ehac508_Supplementary_Data [file ehac508_supplementary_data.zip › 20220818_Supplementarytable_I_EHFlash.docx]

|  | **Right Ventricle** | | **Left Ventricle** | |
| --- | --- | --- | --- | --- |
|  | Before  thrombectomy | After  thrombectomy | Before  thrombectomy | After  thrombectomy |
| Ejection fraction (TTE, %)  **Pressure-Volume loop**  Stroke volume (mL)  End-diastolic volume (mL)  End-systolic volume (mL)  End-systolic pressure (mmHg)  End-diastolic pressure (mmHg)  End-systolic elastance (E_es_, mmHg mL^-1^)  Arterial elastance (E_a_, mmHg mL^-1^)  E_es_ / E_a_ ratio  Stroke work (SW, mmHg mL^-1^)  Potential energy (mmHg mL^-1^)  Pressure volume area (PVA, mmHg mL^-1^)  SW / PVA ratio | 32  37.5  139.0  94.9  37.6  13.9  0.40  1.00  0.39  770.3  1778.3  2548.6  0.30 | 49  41.9  101.0  52.0  16.4  4.6  0.32  0.39  0.81  751.7  425.3  1177.1  0.64 | 50  38.0  76.0  38.0  122.6  5.0  1.24  2.59  0.48  5299.2  6060.8  11360.0  0.47 | 58  41.8  71.8  30.1  103.2  6.4  1.24  2.02  0.61  4427.8  4294.5  8722.3  0.51 |

**Supplementary table 1** Biventricular changes in transthoracic echocardiographic assessment and pressure-volume loops immediately before and after transcatheter thrombectomy.

*E_a_: Arterial Elastance, E_es_: End-systolic elastance, PVA: Pressure Volume Area, SW: Stroke Work, TEE: Transthoracic Echocardiography.*

*The mentioned (volumetric) values present averages which preclude 1:1 correlations.*
